# Two Species Delimitation of *Pseudaulacaspis* (Hemiptera: Diaspididae) Based on Morphology, Molecular Clustering, and Niche Differentiation

**DOI:** 10.3390/insects14080666

**Published:** 2023-07-25

**Authors:** Yunyun Lu, Shuqun Deng, Minmin Niu, Huiping Li, Qing Zhao, Hufang Zhang, Jiufeng Wei

**Affiliations:** 1 College of Plant Protection, Shanxi Agricultural University, Jinzhong 030801, China; lyyun1995@gmail.com (Y.L.); ds360265@163.com (S.D.); niuminmin@nwsuaf.edu.cn (M.N.); zhaoqing86623@163.com (Q.Z.); 2 Technology Center of Taiyuan Custom, Taiyuan 030006, China; lihuipingyy@163.com; 3 Department of Biology, Xinzhou Teachers University, Xinzhou 034000, China

**Keywords:** *Pseudaulacaspis*, species delimitation, morphological traits, molecular clustering, climatic niche, niche overlap, niche comparison

## Abstract

**Simple Summary:**

Two species belonging to the genus *Pseudaulacaspis* MacGillivray, 1921 (Hemiptera: Diaspididae), *Pseudaulacaspis pentagona* (Targioni Tozzetti, 1886) and *Pseudaulacaspis prunicola* (Maskell, 1895) are well-known pests for a great variety of ornamental plants and fruit trees worldwide. Both are notorious pests and significantly similar in morphology characteristics, life cycle, and ecological conditions, making it quite challenging to distinguish between the two species. In this paper, we implemented an integrative approach combining morphological, molecular, and ecological niche analyses in order to species delimitation. Overall, our findings indicate that the results further underpin the notion that the two species are closely related but distinct. We show that the integration of multiple approaches is useful in identifying morphologically similar species of the genus *Pseudaulacaspis*, overcoming the difficulties encountered when using traditional taxonomy alone.

**Abstract:**

*Pseudaucalaspis pentagona* and *P. prunicola* are notorious pests and commonly feed on various ornamental plants and fruit trees worldwide. The two species share many host-plant species, and are similar in morphological characteristics and life cycle, making it difficult to distinguish to distinguish between them. In this study, morphological characteristics, molecular evidence, and ecological niches were used to define these species. We performed PCA analysis on 22 morphological characteristics that allowed the delineation of the species. We then sequenced the COI gene of both species revealing five populations of *P. pentagona* and one population of *P. prunicola*, and the higher support rate could distinguish the two species. We also identified the potential distribution area of the two species based on the MaxEnt niche model, which showed that the degree of niche overlap was high, but that they occupied different niches. Ultimately, we combined three lines of evidence to show that the two species are distinctly different. This study supports species definition using combined morphology, genetics, and ecology and provides a theoretical basis for the effective control of these two pests in the future.

## 1. Introduction

Armoured-scale insects maintain the largest and most specialized position among the dozen or so families currently recognized as the superfamily Coccoidea [1].

Diaspididae are one of the most successful small herbivorous insects with piercing–sucking mouth parts and are major economic pests as they attack and destroy perennial ornamentals and food crops [2,3]. Individuals of most scale insect species are small and cryptic in habit. They are serious economic pests and are among the most invasive insects in the world. The insect is almost ubiquitous among woody plants, but its identification is indeed very difficult, because the adult female is at the only critical stage in which slides can be made and its characteristics can be identified.

The genus *Pseudaulacaspis* MacGillivray, 1921 (Hemiptera: Diaspididae) currently contains 66 species known worldwide, of which the most species were recorded in south-east Asia [4]. The white peach scale *P. pentagona* (Targioni-Tozzetti) (Hemiptera: Diaspididae), is native to eastern Asia, and is a common pest in orchards [5]. It is an extremely invasive species and was first reported in Florida, USA but is now widely distributed in 112 countries located in different climatic zones. *P. pentagona* feeds on a wide range of host plants, including 90 families and 253 genera of host plants and particularly affects plants of the Rosaceae family. [4]. It also attacks fruit trees [6], ornamental plants [7], and wild plants [8], and shows high fecundity on potato plants, as proved by experimental research [9]. It causes very serious damage to peach trees in Turkey and the eastern Mediterranean region [10], and causes serious economic losses to the untreated pear industry in the United States, with annual losses reaching 480,000 dollars [11]. Notably, generations of *P. pentagona* are different in different regions of Turkey, where for example, it has two generations in mountainous areas but three generations in coastal areas [10]. Lu Yunyun et al. used MaxEnt to predict the potential distribution pattern of *P. pentagona* under current and future climatic scenarios based on global occurrences. The results indicated that, in a warming climate, the region of climatic adaptability would be larger than current condition, especially in east Asia and Europe [12].

The white prunicola scale, *P. prunicola* (Maskell) (Hemiptera: Diaspididae), is native to the northern temperate areas of China and Japan and seriously threatens *Prunus* species in temperate areas. Previous literature reported that *P. prunicola* can infect 18 families and 26 genera of plants, including Apocynaceae and Rosaceae [4]. It is widely distributed in eight countries, including China, the United States, and India. It was originally reported to have caused significant damage to Japanese plums in Hawaii. 

In relation to *P. pentagona* and *P. prunicola*, Kawai (1980) and Davison et al. (1983) both advocated for the classification of these as separate species [13,14]. Kawai posited that the distinguishing characteristics between the two female adults are as follows: *P. prunicola* possesses spherical antennae, with two gland spines between the third and fourth lobes that are pointed apically; whereas *P. pentagona* possesses cone-shaped antennae, with one gland spine between the third and fourth lobe that are apically branched. Davidson differentiated the two species based on variations in egg color, host plants, and life history [14]. In terms of their geographical distribution pattern, *P. prunicola* primarily occupies temperate regions and exhibits a life cycle of two generations per year, while *P. pentagona* predominantly thrives in tropical and subtropical regions and undergoes a life cycle of three generations per year. Both species have a wide distribution on various host plants, but *P. pentagona* primarily inflicts damage on *Morus* plants, whereas *P. prunicola* primarily affects *Prunus* plants. Fang-teh Tang posited that *P. prunicola* was not an independent species, but rather a synonym for *P. pentagona*. The evident geographical range of *P. pentagona* extends from Yinchuan in the north to Guangzhou in the south, while the apparent distribution of *P. prunicola* spans from Yinchuan in the north to Liuzhou in the south. Additionally, when considering the host plant, *P. pentagona* is exclusively found on *Morus,* whereas *P. prunicola* may be present on both *Morus* and other plants. Furthermore, in terms of morphological characteristics, variations in the shape of antennae and gland spines on the left and right sides of the same individual represent different types of *P. prunicola* and *P. pentagona* [15].

Species delimitation is the basis of many biological research fields, but it is also a heavy burden for many taxonomists, mainly due to cryptic species [16]. The traditional identification of species relies on morphological traits [17,18]. On its own, this method is limited and can result in incorrect identification due to phenotypic plasticity and genetic variability of certain traits. For scale insects that show extreme sexual dimorphism, identification is generally based on the morphology of adult females [19]. This is, however, difficult to apply in practice, such as in customs phytosanitary scenarios, where samples vary in growth stages and gender, and it can lead to indistinguishability [20].

At present, many studies introduce ecological niches as a factor in biodiversity research [21,22,23]. Different from the concept of a biological species, the ecological species [24,25] emphasizes the species occupying a unique ecological niche, further expanding the concept of a species. The interactions between internal and external factors that promote the differentiation of a biological species can thus be combined to analyze the boundaries between different species. The entire life cycle of a scale insect depends on climatic factors and climate change will affect its life history, physiological characteristics, fecundity, and population dynamics, which in turn will affect their distribution. Models based on climatic niches have been widely used in species distribution studies including animals, plants, and microorganisms. Quantifying the niche differentiation between closer taxa can play a very important role in understanding the pattern of speciation and the dynamics of species evolution.

In the present study, we aimed to distinguish between these two *Pseudaulacaspis* species using a three-pronged approach involving morphological data, molecular data, and ecological niche data.

## 2. Materials and Methods

### 2.1. Sample Collection

Individuals of *P. prunicola* were collected from Yinchuan City in the Ningxia Province; Riverfront Park Linfen City in the Shanxi Province; Taigu County, Jinzhong City in the Shanxi Province; Taiyuan City in the Shanxi Province; Dinagbian County, and Yulin city in the Shaanxi Province. Individuals of *P. pentagona* were collected from Aolys japonica in Nanhu Park, Taizhou District, Fuzhou City, and Fujian Province (Table 1). The adult females were collected from different hosts plants, with three or more specimens at each sample location. All specimens were taken from the branches of host plants. The host plants and specimens were soaked together in 100% alcohol, put into 5 mL cryopreservation tubes, and stored at −20 °C for subsequent operations. It is common practice for taxonomists of scale insects to use whole individuals for DNA extraction due to their small size, while relying on other individuals collected from the same branch to prepare slides for morphological identification. This significantly lowers the precision of morphological identification. For this study, however, species slides were prepared following a new method allowing for the isolation of scale-insect DNA without destroying morphological features (see the following section for more detail). Through this improved kit-extraction method, we could extract the whole genome of scale insects while preserving all morphological characteristics.

### 2.2. DNA Extraction and PCR

We used three single adult whole bodies for DNA extraction from each collection site. From here we chose a single test scale insect and placed it on a clean glass slide. We used 100% EtOH to remove the surface scales, surface bacteria, other attachments, and plant material. We then pricked a hole in the abdomen of the scale insect with a clean ‘000’ insect pin, added 10 µL of proteinase K and 90 µL of Buffer ATL, and incubated the sample in a 1.5 mL microcentrifuge tube for 12 h. Following overnight digestion, individual scale cuticles were removed from the lysate using wide-mouth micropipette tips for subsequent slide mounting. The total DNA was extracted from the lysate using the Qiagen DNeasy Blood & Tissue kit (Qiagen, Valencia, CA, USA), and stored at 4 °C for further use. The remainder of the extraction followed the manufacturer’s specifications for the Qiagen kit.

From the total genomic DNA, PCR was performed to amplify a region of the mitochondrial gene cytochrome oxidase I (COI) gene using the primer pair [26,27]: PcoF1: 5′—CCTTCAACTAATCATAAAAATATYAG—3′; LepR1: 5′ –TAAACTTCTGATGTCCAAAAAATCA—3′. The PCR reactions contained a total volume of 25 μL, which included 12.5 μL 2×Taq MasterMix (With dye) (Coolaber, Beijing, China), 8.5 μL distilled water, 1 μL of each primer, and 2 μL DNA template. A negative control was included for all reactions. To amplify the fragment, we used the T100TM Thermal Cycler (BIO-RAD Laboratories, Inc., Hercules, CA, USA) with the following amplification program: 2 min at 94 °C, then 30 cycles at 94 °C for 40 s, 51 °C for 40 s, and 72 °C for 1 min, followed by incubation for 6 min at 72 °C. The PCR products were examined on a 1.5% agarose gel. Positively amplified products were sent to Tsingke Biotechnology Co., Ltd. (Beijing, China) for Sanger sequencing.

### 2.3. Sequence Analysis and Molecular Systematics

All mtDNA (including the outgroup) were newly sequenced for this study. Raw sequences were analyzed, edited, and aligned using MAFFT v7 [28]. Gblocks0.91b software [29] was used to extract the conserved sites from multiple sequence alignment results. The mtDNA sequences generated in this study are available from the GenBank database with the following accession numbers—*P. prunicola*: OQ941482—OQ941484, OQ941485, OQ942036—OQ942039, OQ942028—OQ942033; *P. pentagona*: OQ933459, OQ941477—OQ941481; *C. aonidum*: OQ943783.

Neighbor-joining (NJ) analysis performed in MEGA11 with 1000 replicates bootstrap [30]. Using jModelTest v0.1.1 under the BIC criterion, we selected the T92 + G + I model as the optimal base substitution model and correlation parameter [31]. Maximum Likelihood (ML) and Neighbor-joining (NJ) trees were constructed in MEGA v11 with 1000 bootstrap replicates to test the reliability of the inferred clades.

### 2.4. Species Occurrence Data

**Figure 1:**
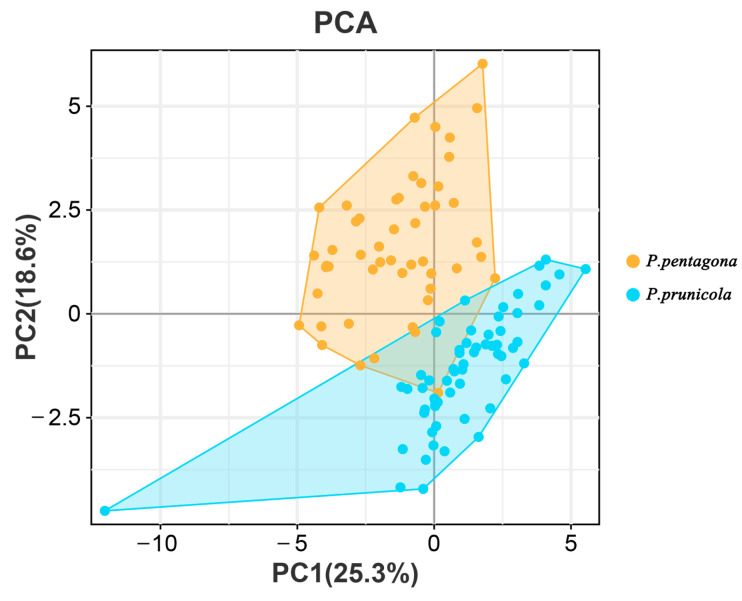
Principal components analysis (PCA) results of 22 morphological traits survey for *P. pentagona* (*n* = 50) and *P. prunicola* (*n* = 60).

Geographical occurrence data of the two species were obtained from the following resources: specimens preserved in the Insect Herbarium of the Plant Protection College of Shanxi Agricultural University; and newly collected fresh specimens for this study. Occurrence data with unclear or inaccurate information were eliminated. Finally, we retained occurrence data for *P. pentagona* (*n* = 47 points) and *P. prunicola* (*n* = 24 points) (Table S1) (Figure 1). The geo-coordinates for each site were retrieved using Google Maps (https://www.google.com/maps/) (accessed on 23 June 2022). In the process of sample collection, sampling will face different collection intensities [32]. To eliminate sample bias and remove spatial autocorrelation, we established grids with an area of 25 km^2^ out of the data in the study area. In a grid with multiple occurrence data, we randomly selected an occurrence data point [33]. After processing, the occurrence data for *P. pentagona* was reduced to 44, while the occurrence data for *P. prunicola* was reduced to 24 (Table S2).

### 2.5. Climatic Variables

Temperature and precipitation factors affect insect species distribution patterns [34]. Thus, to quantify the impact of these key factors on the study taxa, we downloaded 19 climatic variables (Bio1-Bio19) from the WorldClim Database (http://www.worldclim.org) (accessed on 23 June 2022) [35]. These climatic variables were compiled by different measuring agencies around the world and recorded using monthly, quarterly, and annual ambient temperature and precipitation and seasonal differences from 1950 to 2000 including the average, minimum, and maximum values. The common resolution of all used climatic variables was chosen as 2.5 arc min. It is known that the incorporation of more environmental factors in a model can result in strong multicollinearity between climatic variables rendering the model overfit [36], which will reduce model performance. To eliminate this, we used the Pearson correlation coefficient to analyze the pairwise correlations of the 19 environmental variables using SPSS v17 and only retained variables with Pearson correlation coefficients less than 0.8 (|r| < 0.8) [37]. Finally, the climatic variables Bio2 (mean diurnal temperature difference), Bio3 (isothermality), Bio8 (mean temperature of the wettest season), and Bio15 (precipitation seasonality) were used for predictive modeling (Table S3).

### 2.6. Morphometric Analyses

All specimens used to extract total genomes (*P. pentagona* N = 6, *P. prunicola* N = 15) and amplify COI fragments were slidemounted for morphological identification. The rest of the specimens came from the Insect Herbarium of Plant Protection, Shanxi Agricultural University. Species were slide-mounted using the method described by Benjamin B. Normark et al. (2019) [2]. The preparation of the specimen slides includes three procedures of staining, dehydration, and slide production: after total genome extraction, the whole cuticles were stained for five minutes in a 1% acid fuchsin solution; 75% ethanol, 5 min; 100% ethanol, 5 min; xylene solution, 5 min. Each cuticle was mounted separately with Canada balsam in the center of the slide, covered with a coverslip. Macroduct and gland spine numbers are important taxonomic morphological characteristics of *Pseudaulacaspis* [38]. To obtain more comprehensive information about *P. pentagona* and *P. prunicola*, we examined 110 specimens (*P. pentagona* N = 50, *P. prunicola* N = 60) preserved in the Insect Herbarium of Plant Protection College of Shanxi Agricultural University. A summary of species sampling locations used for morphometric measurements is shown in Table 2. We selected 19 traits for statistical analysis and measurement, including the number of disc pores, the number of dorsal macroducts in abdomen segment Ⅱ, the number of dorsal macroducts in abdomen segment Ⅲ, the number of dorsal macroducts in abdomen segment Ⅳ, the number of dorsal macroducts in abdomen segment Ⅴ, the number of dorsal macroducts in abdomen segment Ⅵ, the number of perivulvar pores, the number of gland spines in the first space, first space gland spine branched or not, the number of gland spines in the second space, second space gland spine branched or not, the number of gland spines in the third space, third space gland spine branched or not, the number of gland spines in the fourth space, fourth space gland spine branched or not, insect body length, insect body width, distance of anal opening to L1, L1 width, distance between L1, and distance between antennae. We also included three calculation parameters: the ratio of insect body width: length, the ratio of anal opening to L1: insect body length, and the ratio of width between L1: L1 width (Table S4). All morphological work was completed using a Murzider microscope and accompanying measurement software. Using the R packages “factoextra” and “factomineR” [39,40], principal component analysis (PCA) was used to identify phenotypic clustering.

### 2.7. Climatic Niche Modeling

Over the past few decades, species distribution models have been widely used for estimating the temporal and spatial distribution patterns of species, the planning and regulation of harmful insect prevention areas, the early warning of alien species invasion [41], the modes and pathways of disease transmission [42,43] the impact of global climatic changes on species distribution or diversity patterns [44] and evolutionary biology [45]. Recently, many studies have been published on closely related species delimitation using multiple approaches including climatic niche modeling. Multiple species distribution models have also been created and implemented based on different principles, such as GRAP (Genetic Algorithm for Rule-set Prediction) [46], ENFA (Ecological Niche Factor Analysis) [47], GAM (Generalized Additive Model) [48], BIOCLIM (Bioclimatic envelope) [49], DOMAIN [50], CLIMEX [51] and MaxEnt (maximum entropy) [52]. MaxEnt fits complex models using smaller datasets, especially presence-only datasets and prevents model complexity from increasing beyond what the empirical data support, thereby stimulating the distribution of species under more realistic habitat conditions. MaxEnt also relies on stable calculations and conservative results, which can be constrained based on environmental conditions based on record locality and has become one of the mostly widely applied software for climatic niche modeling [53]. The maximum entropy algorithm (MaxEnt 3.4.3k, http://www.cs.priceton.edu/~schapire/maxent/) (accessed on 26 June 2022) was used to map the distribution of the two species.

In general, the MaxEnt software default parameters were set and applied in some research, and relevant results demonstrated that the model complexity affects model performance [53]. To avoid model overfitting, while simultaneously increasing predictive power, we applied the R package “ENMeval” (R v 3.6.3) to select the ideal pairing of two crucial MaxEnt parameters, the value of the regularization multiplier (RM) and the combination of feature classes (FCs) [54]. We set the RM value rage to 0.5–4, with every class increment set to 0.5. The 8 FCs combination was as follows: (i) Linear (L); (ii) Linear (L) and Quadratic (Q); (iii) Linear (L), Quadratic (Q) and Hinge (H); (iv) Linear (L), Quadratic (Q), Hinge (H), and Product (P); (v) Linear (L), Quadratic (Q), Hinge (H), Product (P), and Threshold (T); (vi) Quadratic (Q), Hinge (H) and Product (P); (vii) Quadratic (Q), Hinge (H), Product (P), and Threshold (T); (viii) Hinge (H), Product (P) and Threshold (T). The “checkerboard2” method was used to calculate the Akaike information criterion coefficient (AICc), and the final MaxEnt model adopted the lowest delta AICc scores to perform.

We set each model so that 75% of the distribution data was allocated for training data and 25% for testing data. To further ensure the accuracy of the prediction results and prevent random errors, 10,000 background points and 10 repetitions were performed. Based on the 10th percentile replicate training presence logistic threshold, the continuous maps of *P. pentagona* and *P. prunicola* were transformed into binary maps of suitability or probability [55], in which pixels are classified as “adaptive/presence” and “non-adaptive/absence”. The suitable habitat area was divided into three levels for clarity: (1) low suitable habitat: 0.25–0.50; (2) moderately suitable habitat: 0.50–0.75; (3) highly suitable habitat: 0.75–1.00.

### 2.8. Model Evaluation

We adopted the pROC (partial receiver operating characteristic) method of model evaluation to evaluate the performance of the model to avoid the disadvantages of the ROC (partial operating characteristic) characteristic curve, namely, equal weighting of omission and commission errors and an AUC that cannot provide information on the spatial distribution of model errors [56]. The NicheToolbox site was used to validate pROC for model performance with 1000 replicates and E = 0.05 (http://shiny.conabio.gob.mx:3838/nichetoob2/) (accessed on 28 June 2022) [57].

### 2.9. Niche Comparison

To investigate whether climatic niches differ between *P. pentagona* and *P. prunicola*, we used the ‘Ecospat’ package in (R v 3.6.3) to maximize the isolation, quantification, and comparison of climatic and spatial environmental conditions in the study area [58]. Tools for supporting spatial analysis and the modeling of species niches and distributions are included in ‘Ecospat’ and allow for the quantification of niche overlaps between the two species [59]. For niche changes, the following three quantitative indexes are most commonly used: the niche overlap index (D), niche equivalence, and niche similarity. The D value (range from 0–1) represents the degree of ecological niche overlap of the two species, where larger D values suggest higher niche overlaps.

## 3. Results

### 3.1. Statistical Analyses of the Morphological Dataset

In the PCA analysis results of 22 morphological traits of *P. pentagona* (N = 50) and *P. prunicola* (N = 60), the first component (PC1) explained 25.3% of the total variance, and the second component (PC2) explained 18.6% (Figure 1). This dataset separated *P. pentagona* from *P. prunicola*, with only a small amount of overlap in samples from the two species. Based on principal component loading scores, we interpreted dimension 1 as a representation of gland spines type about two species delimitation. The principal component loading scores of variables including first space gland spine branched or not, second space gland spine branched or not, third space gland spine branched or not and fourth space gland spine branched or not are all greater than 0.15. Dimension 2 as a representation of related indicators of lobe1. The principal component loading scores of variables including distance between L1 and the ratio of width between L1: L1 width are all greater than 0.15. Indeed, paying attention to the results of PCA, the above six variables could best distinguish the morphological separation of *P. pentagona* and *P. prunicola* (Table 3).

### 3.2. Phylogenetic Relationship

**Figure 2:**
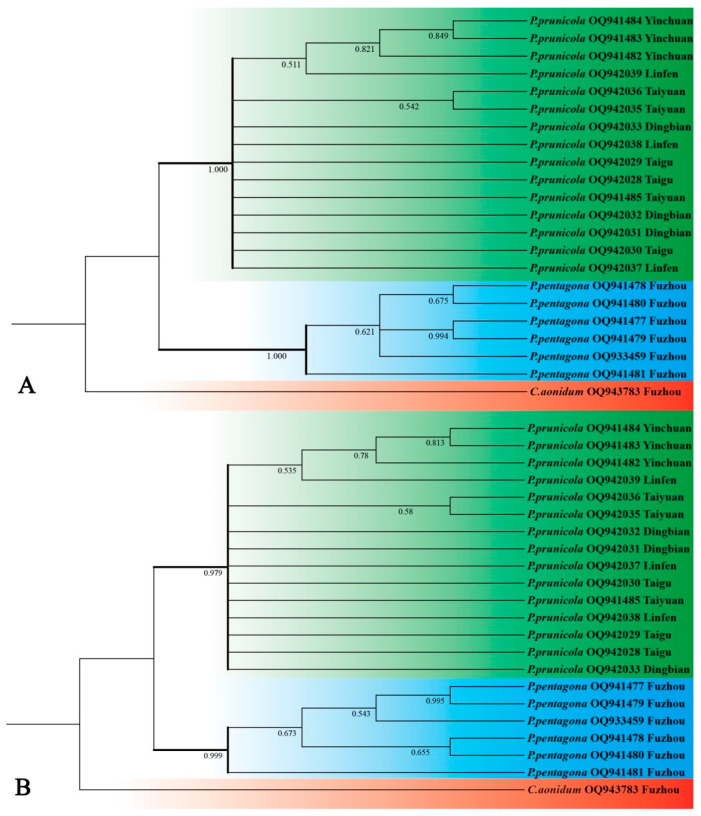
(**A**). The Maximum likelihood (ML) analysis based on mtCOI sequences of the two species in *Pseudaulacaspis*; and (**B**) the Neighbor-Joining (NJ) analysis based on mtCOI sequences of the two species in *Pseudaulacaspis*. Numbers on the nodes are posterior probability (PP).

The COI gene sequence results showed high-quality nucleotide sequences of 660–680 bp in length. The subsequent NJ and ML analyses showed identical tree topologies. Both datasets resulted in two lineages, representing two statistically monophyletic clades corresponding to *P. pentagona* and *P. prunicola* (Figure 2). The nodes of the two monophyletic groups showed high support (97.9–100% bootstrap support), confirming the morphological identification.

### 3.3. ENMeval Optimized Parameters and Model Performance

Based on the sampled distribution points and climate data, we predicted the current potential distribution of *P. pentagona* and *P. prunicola*. The final model parameter fitting results generated by the R package ENMeval are shown in Figure S1 and Table S5. The regularization multiplier value was set to 1 and the feature combinations were selected as LQH (Linear, Quadratic, Hinge) for *P. pentagona* in the final program. For *P. prunicola*, the regularization multiplier value was set to 1 and the feature combinations were selected as LQP (Linear, Quadratic, Product).

The model performance for the two species demonstrated significant predictive power based on the partial ROC tests (mean value AUC for *P. pentagona*: 0.8870003; mean value AUC for *P. prunicola*: 0.8111001), and the distribution of the AUC ratio AUCpartial/AUCrandom was significantly higher than random expectation, demonstrating the high performance of our model (*p* < 0.0001) (Figure S2).

### 3.4. Impact Analysis of Key Climatic Variables

The following table (Table 4) lists the estimates of the relative contributions of environmental variables to the MaxEnt model. Temperature and precipitation were critical factors in the growth, development, and survival of these insects. However, when examined closely, temperature was more important to the growth and potential distribution of these two insects than precipitation. For *P. pentagona*, isothermality (Bio3) had the highest contribution rate (35.4%) to the model, and the mean temperature of the wettest quarter (Bio8) was the second largest contributor (34.8%). Mean diurnal range (Bio2) and precipitation seasonality (Bio15) also contributed 11.9% and 18.2% respectively. For *P. prunicola*, the mean temperature of the wettest quarter (Bio8) had the highest contribution rate (45.7%) to the model. Second, mean diurnal range (Bio2), isothermality (Bio3), and precipitation seasonality (Bio15) contributed 24.6%, 23.1%, and 4.6%, respectively.

### 3.5. Comparison of Current Potential Distribution Areas and Niche

**Figure 3:**
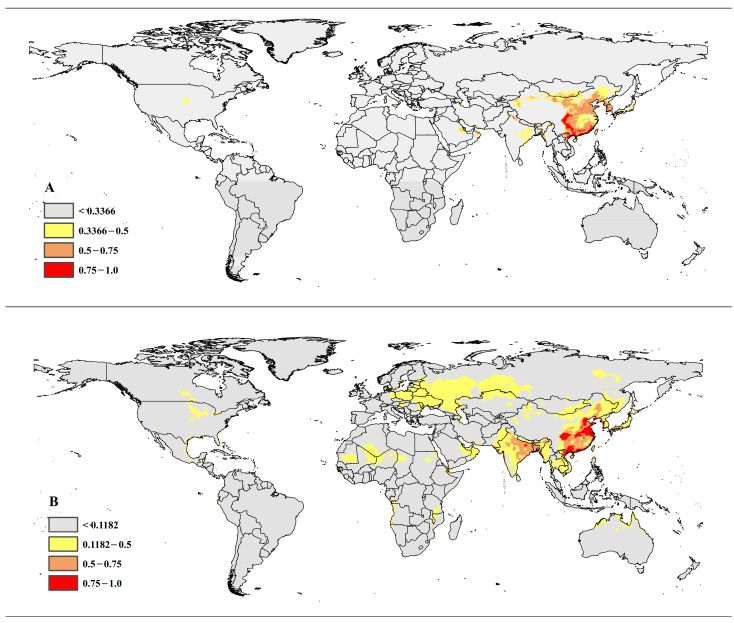
(**A**) The potential distribution map for *P. pentagona*; and (**B**) the potential distribution map for *P. prunicola*; Red, High suitability areas; Bown, Moderate suitability areas; Yellow, Low suitability areas; Gray, Unsuitable areas.

From a global perspective, the potential distribution areas of *P. pentagona* and *P. prunicola* are concentrated in the eastern part of the Asian plate, while some areas in Europe and Africa are suitable for *P. prunicola* survival (Figure 3). The high-suitability areas for *P. pentagona* are mainly concentrated in the eastern part of the Shandong Peninsula, with star points in Shaanxi, Sichuan, Guizhou, Chongqing and other places in southwest China, the coastal areas of Guangxi, Guangzhou, and Fujian, the coastal areas of northwestern Taiwan, the coastal areas of northeastern Vietnam, southern Iran, the northern coastal area of Oman and parts of northern India. The Liaoning Peninsula, Shandong Peninsula, North China Plain and most of East China, Zhejiang, Fujian, and Guangdong coastal areas, the entire territory of Guangxi, Taiwan Island and the northern coastal area of Hainan Island, the eastern coastal area of the Korean Peninsula, northern Vietnam, the western coastal area of Myanmar, and the eastern and the western coast and northern parts of India are also very suitable for *P. prunicola* survival. The results show that there is a significant risk of colonization of *P. pentagona* and *P. prunicola* to the east of the Huanyong Line (Tengchong-Heihe Line) within the territory of China, including northeast China, north China, east China, central China, south China and southwest China (Figure 3). Similarly, the two pests have the same potential for colonization of the border between China and Mongolia, parts of Xinjiang, the Korean Peninsula and southern Japan. Additionally, *P. prunicola* has the potential to colonize European countries including Ukraine, Belarus, Poland, eastern Germany, Latvia, and Estonia, Russia’s eastern region near the Black Sea and where Russia borders Kazakhstan, as well as the Indian peninsula and parts of southeast Asia.

**Figure 4:**
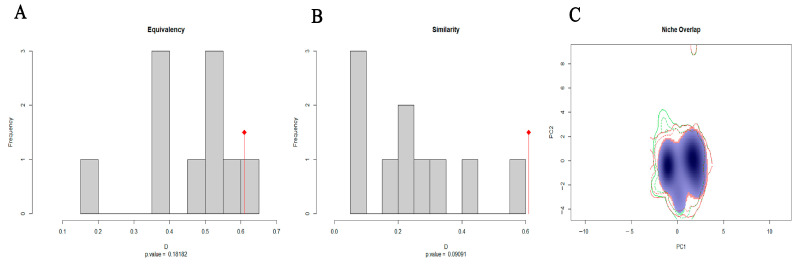
Climatic niche differentiation between *P. pentagona* and *P. prunicola*: (**A**) results of climatic niche equivalency; (**B**) results of climatic niche similarity tests; and (**C**) species density of *P. pentagona* and *P. prunicola* along two climatic gradients.

Niche comparison revealed a 60.94% (Schoener’s D = 0.6094) niche overlap between *P. pentagona* and *P. prunicola*. We also analyzed niche similarity and niche equivalence for the two species, where niche similarity illustrates the similarity of the relative distribution of environmental conditions over a long time (Figure 4) and niche equivalence assesses whether the environmental conditions of the different species are the same. For paired comparative niches, the null hypothesis of niche equivalence was rejected, indicating that the climatic niches of the two species pairs were significantly different. For the niche similarity test, our results suggest that the niches of *P. pentagona* and *P. prunicola* contain similar but not equivalent environmental conditions. Based on the above evidence, we suggest that *P. pentagona* and *P. prunicola* are ecologically closely related, but that they do represent different species.

### 3.6. Redescription

Morphological terminology follows Miller and Davidson (2005) [60]. For the illustrations, the dorsum is shown on the left side and the venter on the right. The following abbreviations are used for the pygidial lobes: medial lobe (L1), second lobe (L2), third lobe (L3) and fourth lobe (L4).

#### 3.6.1. Pseudaulacaspis Pentagona (Figure 5)

Material: 4 adult female: China, Yunnan, Jinggu, on *Euphorbia pekinensis* Rupr., Fang-teh Tang; 3 adult female: China, Yunan, Kunming, on *Phoebe zhennan* S. Leeet, F.N.Wei, 5.1975, Fang-teh Tang; 2 adult female: China, Nanning, on *Hbiscus mutabilis* Linn., 28.9.1977, Fang-teh Tang; 3 adult female: China, Wuhan, on *Trachycarpus fortunei* (Hook.) H.Wendl., Fang-teh Tang; 3 adult female: China, Fuzhou, on *Morus alba* L., Fang-teh Tang; 2 adult female: China, Sichuan, Xichang, on *Camellia sinensis* (L.) O. Ktze., Fang-teh Tang; 1 adult female: China, Dalian, on *Fraxinus rhynchophylla* Hance, Fang-teh Tang; 1 adult female: China, Shantou, on *Ricinus communis* L., 4.11.1977, Fang-teh Tang; 1 adult female: China, Kunming, Wenquan, 6.9.1977, Fang-teh Tang; 1 adult female: China, Sichuan, Emeishan, on *Cycas revoluta* Thunb., 14.9.1976, Fang-teh Tang; 3 adult female: China, Beijing, The Summer Palace, on *Prunus armeniaca* L., 20.5.1972, Fang-teh Tang; 7 adult female: China, Tianjin, on *Ailanthus altissima*, 21.11.1979, Fang-teh Tang; 1 adult female: China, Taiyuna, on *Osmanthus* sp., Fang-teh Tang; 2 adult female: China, Zhejiang, Huangyan, on *Ricinus communis* L., 6.1972, Fang-teh Tang; 1 adult female: China, Guangdong, Shantou, on *Ricinus communis* L., 4.11.1977, Fang-teh Tang; 1 adult female: China, Sichuan, Emeishan, on *Triadica sebifera* (Linnaeus) Small, 14.9.1976, Fang-teh Tang; 1 adult female: China, Zhejiang, Hangzhou, on *Ligustrum sinense* Lour., 5.1979, Fang-teh Tang; 1 adult female: China, Tianjin, on *Juglans regia* L., Fang-teh Tang; 1 adult female: China, Zhejiang, Hangzhou, on *Morus alba* L., 1.4.1979, Fang-teh Tang; 2 adult female: China, Tianjin, on *Styphnolobium japonicum* (L.) Schott, Fang-teh Tang; 4 adult female: China, Gangu, Lanzhou, on *Prunus persica* L., 9.6.1974, Fang-teh Tang; 2 adult female: China, Guangdong, Gunagzhou, on *Morus alba* L., Fang-teh Tang; 3 adult female: China, Sichuan, Chengdu, on *Paulownia Sieb*.et Zucc., Fang-teh Tang; 1 adult female: China, Anhui, Maanshan, on shrub, 20.4.1982, Fang-teh Tang; 1 adult female: China, Sichuan, Chengdu, Wuhou Shirne, on *Paulownia Sieb*.et Zucc., Fang-teh Tang; 3 adult female: China, Fujian, Shuyang, on *Prunus persica* L., 11.9.1977, Fang-teh Tang; 1 adult female: China, Shanxi, Taiyuan, on *Amygdalus triloba*, 15.5.1969, Fang-teh Tang; 5 adult female: China, Tianjin, on *Ailanthus altissima*, Fang-teh Tang; 4 adult female: China, Yunnan, Mengzi, on *Prunus persica* L. 20.9.1974, Fang-teh Tang; 3 adult female: China, Sichuan, Emeishan, on *Fraxinus chinensis* Roxb., 14.9.1976, Fang-teh Tang; 3 adult female:China, Shanxi, Taiyuan, on *Osmanthus* sp., 7.4.1983, Fang-teh Tang; 2 adult female:China, Guangxi, Nanning, on *Hibiscus mutabilis* L., 28.9.1977, Fang-teh Tang; 2 adult female: China, Liaoning, Dalian, on *Fraxinus chinensis*, 15.8.1976, Fang-teh Tang; 1 adult female: China, Tianjin, on *Ailanthus altissima*, 3.1981, Fang-teh Tang; 3 adult female: China, Guangxi, on *Hibiscus mutabilis* L. 28.9.1977, Fang-teh Tang; 5 adult female: China, Yunnan, West Mountain of Kunming, on *Lauraceae* Juss., 3.9.1977, Fang-teh Tang.

**Figure 5:**
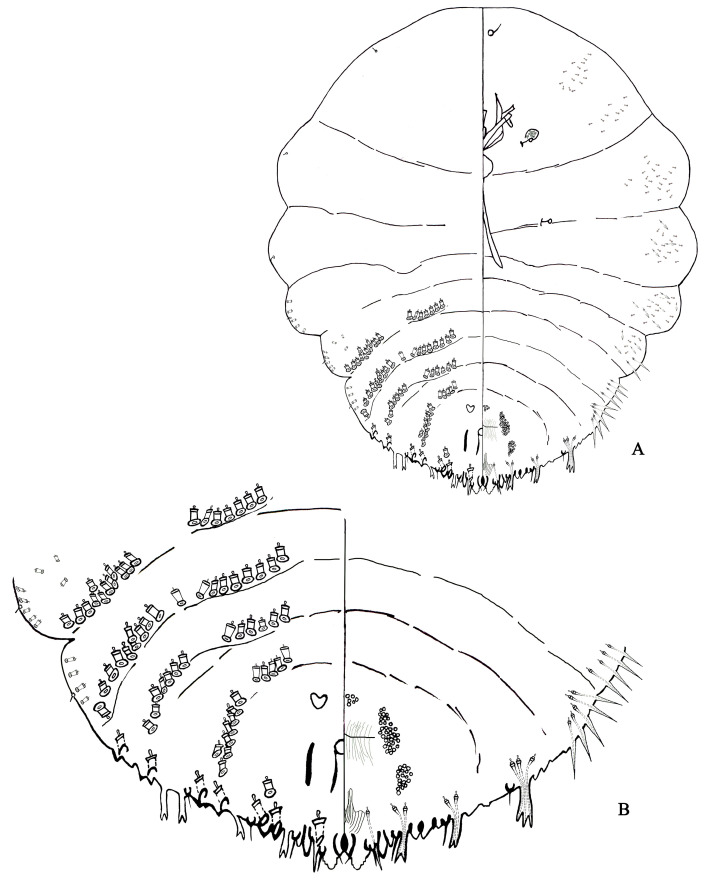
*Pseudaulacaspis pentagona*, adult female: (**A**) body; and (**B**) pygidium.

Diagnosis (Figure 5). Description, *n* = 79. Adult female not pupillarial. Scale cover *grayish* white, nearly round, with highly convex humped shape, and with orange exuviae at one side of scale cover. Broadest at metathorax or abdominal segment I. Body outline gyroscopic type, body membranous except for pygidium.

Cephalothorax. Antenna simple, with 1 short seta, distance between antenna 22–45 μm; anterior spiracle with 5–23 disc pores, each pore normally 3-locular, posterior spiracle without pores.

Pygidium (Figure 5). Adult female with 4 or 5 pairs of lobes, fifth lobes absent or small. Pygidium broadly rounded. L1 rounded apically, projecting the margin of pygidium, inner margins nonparallel, joined by U-shaped, sclerotized yoke, separated by space 0.37–0.56 times width of median lobe, with a pair of setae between L1, each margin with notches, 1–4 with lateral notches, 1–2 with medial notches; L2 bilobed, inner lobule noticely smaller than L1, outer lobule minute or lacking, rounded apically, without notches; L3 bilobed, inner lobule equal with outer lobule, without notch, inner lobule pointed apically, outer lobule rounded or pointed apically; L4 simple, dentation apically.

Gland spines. One pair of setae prensent between the L1. Long seta usually 1 present in first space, second space, third space and fourth space. Gland spines, longer than lobes, usually 1 prensent in first space, second space and third space, brached apically. In fourth space, 1–4 present in fourth space, One brached apically, others occasionally brached apically.

Ducts. Dorsal macroducts 2-barred, barrel-shaped or slightly more elongate, 1 size on pygidium. Without marginal macroduct between L1. Between L1 1 marginal macroduct, L2, L3, L4 have two marginal macroducts respectively. With 7 marginal macroduct on each side.Dorsal macroducts in submarginal and submedial areas of pygidium similar to those in marginal area: 0–1 in submarginal area of abdominal segment VI; 2–9 in submarginal area and 2–8 in submedial area of abdomen segment V. 3–9 in submarginal area and 3–8 in submedial area of abdomen segment IV; 4–12 in submarginal area and 4–9 in sub-medial area of abdomen segment III; 6–14 in submarginal area and 3–9 in sub-medial area of abdomen segment II.

Anal opening. Near oval, 12–15 μm in diameter, positioned 158–200 μm from the base of L1, near the 1/2 of the base of pygidium. Perivulvar pores in 5 groups, 12–24 in median group, 26–37 in the anterolateral group, 23–37 in the posterolateral group. 71–98 pores on each side of body.

Remark. The third space usually has 1 gland spine on the pygidium; the second, third, or fourth spaces has 1 bifid or trified gland spine.

#### 3.6.2. Pseudaulacaspis Prunicola (Figure 6)

Material: 1 adult female: China, Zhejiang, Hangzhou, on *Prunus subg.* Cerasus sp., 2.4.1969, Fang-teh Tang; 1 adult female: China, Zhejiang, Hangzhou, on *Osmanthus* sp., 2.4.1969, Fang-teh Tang; 1 adult female: China, Zhejiang, Hangzhou, on *Osmanthus* sp., 30.3.1969, Fang-teh Tang; 9 adult female: China, Jiangsu, Suzhou, on *Prunus mume* Siebold & Zucc., 20.10.1980, Fang-teh Tang; 1 adult female: China, Sichuan, Emeishan, on *Eucalyptus saligna* Smith, 14.9.1976, Fang-teh Tang; 1 adult female: China, Sichuan, Emeishan, on *Prunus persica* L., 14.9.1976, Fang-teh Tang; 1 adult female: China, Sichuan, Chengdu, on *Osmanthus fragrans* (Thunb.) Loureiro, 24.8.1977, Fang-teh Tang; 1 adult female: China, Yunnan, on *Triadica sebifera* (Linnaeus) Small, 19.10.1978, Fang-teh Tang; 1 adult female: China, Yunan, on *Sapium discolor* (Champ. ex Benth.) Muell.-Arg., 28.7.1978, Fang-teh Tang; 1 adult female: China, Yunan, on *Ligustrum lucidum* Ait., 26.9.1978, Fang-teh Tang; 1 adult female: China, Yunan, on *Camellia sinensis* (L.) O. Ktze., 7.7.1975, Fang-teh Tang; 3 adult female: China, Neimenggu, Baotou, on *Prunus* spp. 18.5.1981, Fang-teh Tang; 2 adult female: China, Yunan, Kunming, on *Rhododendron simsii* Planch., 8.1973, Fang-teh Tang; 1 adult female: China, Gansu, Lanzhou, on *Ulmus pumila* L., 8.6.1974, Fang-teh Tang; 1 adult female: China, Yunnan, Kunming, on *Firmiana simplex* (Linnaeus) W. Wight, 4.9.1976, Fang-teh Tang; 5 adult female: China, Shanxi, Taigu, on *Periploca sepium* Bunge, 28.5.1973, Fang-teh Tang; 1 adult female: 12 adult female: China, Shanxi, Taiyuan, on Cinnamomum camphora (L.) presl, 15.5.1969, Fang-teh Tang; 12 adult female: China, Zhejiang, Huangyan, on Prunus persica L., 25.3.1969, Fang-teh Tang; 7 adult female: China, Shanxi, Taiyuan, on *Amygdalus triloba*, 15.5.1969, Fang-teh Tang; 8 adult female:China, Shanxi, Taiyan, on *Prunus persica* L., 3.3.1965, Fang-teh Tang; 7 adult female: China, Fujian, Shaxian, 4.9.1977, Fang-teh Tang; 8 adult female: China, Guangxi, on *Triadica sebifera* (Linnaeus) Small, 7.1982, Fang-teh Tang; 5 adult female: China, Shanxi, Taiyuan, Jinci Temple, on *Prunus cerasifera ‘Atropurpurea’*, 29.6.1973, Fang-teh Tang; 5 adult female: China, Shanxi, Taiyuan, on *Amygdalus persica ‘Duplex’*, 16.6.1965, Fang-teh Tang; 1 adult female: China, Guangdong, Guangzhou, on *Phoebe zhennan* S. Lee et F. N. Wei, 27.7.1976, Fang-teh Tang; 1 adult female: China, Zhejiang, Wuyi, 23.8.1976, Fang-teh Tang; 1 adult female: China, Neimenggu, Baotou, on *Prunus* spp., 18.5.1981, Fang-teh Tang; 11 adult female: China, Ningxia, on *Prunus persica* L., 30.9.1983, Fang-teh Tang; 8 adult female: China, Shanxi, Taigu, Shanxi Agricultural University, on *Prunus persica* L., 11.3.1981, Fang-teh Tang; 14 adult female: China, Ningxia, on *Amygdalus triloba*, 29.6.1983, Fang-teh Tang; 3 adult female: China, Shanxi, Taigu, on *Prunus persica* L., 11.3.1981, Fang-teh Tang; 5 adult female: China, Shanxi, Taiyuan, on *Syringa oblata* Lindl., 11.4.1983, Fang-teh Tang; 3 adult female: China, Neimenggu, Baotou, on *Amygdalus davidiana*, 22.7.1983, Fang-teh Tang; 5 adult female:China, Ningxia, on *Prunus* spp., 20.9.1974, Fang-teh Tang; 3 adult female: China, Ningxia, on *Prunus persica* L. 30.7.1983, Fang-teh Tang; 9 adult female: China, Neimengu, Baotou, on *Prunus* spp., 18.5.1981, Fang-teh Tang; 2 adult female: China, Ningxia, Yinchuan, on *Prunus armeniaca* L., 4.5.1961, Fang-teh Tang; 9 adult female: China, Ningxia, on *Prunus mume*, 27.9.1983, Fangteh Tang; 14 adult female: China, Shanxi, Taiyuan, on *Prunus cerasifera ‘Atropurpurea’*, 11.4.1983, Fang-teh Tang; 9 adult female: China, Shanxi, Taiyuan, on *Amygdalustriloba*(Lindl.)Ricker, 25.9.1981, Fang-teh Tang; 9 adult female: China, Ningxia, on *Prunus armeniaca* L., 26.9.1983, Fang-teh Tang; 1 adult female: China, Ningxia, on *Prunus armeniaca* L., 21.10.1977, Fang-teh Tang; 7 adult female: China, Ningxia, on *Prunus persica* L., 28.10.1983, Fang-teh Tang; 1 adult female: China, Ningxia, on *Prunus persica* L., 16.10.1983, Fang-teh Tang; 6 adult female: China, Shanxi, Taiyuan, on *Prunus persica* L., 1.11.1982, Fang-teh Tang; 6 adult female: China, Tianjin, on *Prunus sargentii* Rehder, 3.1981, Fang-teh Tang; 11 adult female: China, Gansu, Lanzhou, on *Prunus armeniaca* L., Fang-teh Tang; 1 adult female: China, Tianjin, on *Ficus microcarpa* L. f., 3.1981, Fang-teh Tang; 5 adult female: China, Gansu, Lanzhou, on *Ulmus pumila* L., 9.6.1974, Fang-teh Tang; 15 adult female: China, Gansu, Lanzhou, on *Prunus armeniaca* L., 8.6.1974, Fang-teh Tang; 3 adult female: China, Ningxia, 29.10.1983, Fang-teh Tang; 1 adult female: China, Shanxi, Taiyuan, on *Amygdalus persica ‘Duplex’*, 16.6.1965, Fang-teh Tang; 2 adult female: Shanxi, Yunan, Kunming, Wenquan, 6.9.1977, Fang-teh Tang; 4 adult female:China, Guangxi, Nanning, on *Hibiscus mutabilis* L., 28.9.1977, Fang-teh Tang; 1 adult female: China, Shanxi, Taigu, Shanxi Agricultural University, on *Prunus persica* L., 9.4.1983, Fang-teh Tang; 4 adult female: China, Tianjin, on *Paulownia fortune*, 12.1979, Fang-teh Tang; 6 adult female: China, Tianjin, on *Ailanthus altissima*, 21.11.1979, Fang-teh Tang; 9 adult female: China, Yunnan, West Mountain of Kunming, on *Lauraceae* Juss., 3.9.1977, Fang-teh Tang; 2 adult female: China, Tainjin, on *Ailanthus altissima*, 3.1981, Fang-teh Tang; 2 adult female: China, Tainjin, on *Styphnolobium japonicum*, 3.1981, Fang-teh Tang; 6 adult female: China, Taiyuan, on *Amygdalus persica ‘Duplex’*, 16.6.1965, Fang-teh Tang; 6 adult female: China, Guangxi, Wuzhou, on *Ricinus communis* L., on 10.10.1977, Fang-teh Tang; 3 adult female: China, Sichuan, Xichang, on *Ricinus communis* L., on 10.2.1982, Fang-teh Tang; 11 adult female: China, Zhejiang, Wuyi, on *Vernicia fordii*, 31.7.1972, Fang-teh Tang.

**Figure 6:**
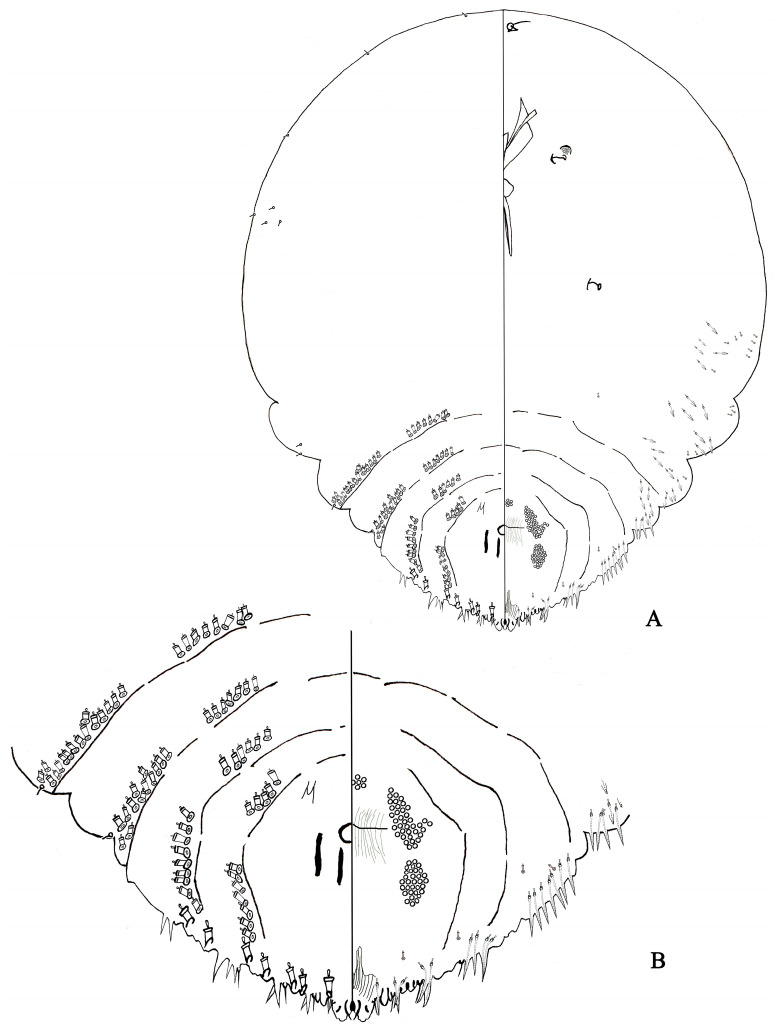
*Pseudaulacaspis prunicola*, adult female: (**A**) body; and (**B**) pygidium.

Diagnosis (Figure 6). Description, *n* = 293. Adult female not pupillarial. Scale cover white, with highly convex humped shape, and with orange exuviae at anterior end. Broadest at metathorax. Body outline gyroscopic type, body membranous except for pygidium.

Cephalothorax. Antenna simple, with 1 short seta, distance between antennae 18.34–40.34 μm; anterior spiracle with 12–50 disc pores, each pore normally 3-locular, posterior spiracle without pores.

Pygidium (Figure 6). Pygidium broadly rounded. L1 rounded apically, inner margins nonparallel, joined by narrow, U-shaped, sclerotized yoke, separated by space nearly equal to width of L1, with a pair of strong setae between L1, inner and outer margin with 1–2 notches; L2 bilobed, inner lobule smaller than L1, outer lobule smaller, rounded apically, without notches; L3 represent by a pair of broad, acuminate, membranous, or sclerotized projections from the body margin.

Gland spines. Long seta usually 1 present in first space, second space, third space and fourth space. Absent between L1, gland spines, longer than lobes, 1 between L1 and L2, pointed apically, usually 2 present second space, 2–4 present third space and 2–10 prensent in fourth space. Gland spines without branches.

Ducts. Dorsal macroducts 2-barred, barrel-shaped or slightly more elongate, 1 size on pygidium. With 7 marginal macroduct on each side. Between L1 1 marginal macroduct, L2, L3, L4 have two marginal macroducts respectively. Dorsal macroducts in submarginal and submedial areas of pygidium similar to those in marginal area: 0–4 in submarginal area of abdominal segment VI; 4–7 in submarginal area and 4–5 in submedial area of abdomen segment V. 5–9 in submarginal area and 4–7 in sub-medial area of abdomen segment IV; 7–14 in submarginal area and 5–7 in sub-medial area of abdomen segment III; 7–17 in submarginal area and 6–9 in sub-medial area of abdomen segment II.

Anal opening. Near oval, 15–20 μm in diameter, positioned 146–152 μm from the base of L1, near the 1/2 of the base of pygidium. Perivulvar pores in 5 groups, 11–18 in median group, 21–45 in the anterolateral group, 26–32 in the posterolateral group. 49–143 pores on each side of body.

Remark. The third space usually has 2 or more gland spines; the second, third, and fourth spaces usually only have simple gland spines.

## 4. Discussion

Phenotype is the external expression of biological traits, while genotype and environment are two factors that determine phenotype [61]. These three aspects are both synergistically related and mutually influencing. In recent years, it is common practice to assemble multidimensional evidence for a detailed delimitation of species. This provides new insights into the current division of many species in which morphology is not readily identifiable [62], the identification of cryptic species, and the delineation of infraspecific species [63,64]. In this study, we used multi-dimensional perspectives including morphological characteristics, molecular methods, and ecological niche analysis to delineate between the two scale insect species.

A clear definition of a species is the basis of most biological experiments, which is vital for the proper selection of subjects [65]. If the species identification is wrong, it renders all other work useless. Nowadays species identification still relies heavily on morphology, but this method has limitations. Morphological identification is usually only valid for specific life stages or sexes. For example, armored scale insects are generally identified by the morphology of adult female individuals. Males have wings, are active, have no mouthparts, do not feed, and are short-lived, making them difficult to collect [2]. Here, we quantified the morphological characteristics commonly used for the classification of armored scale insects, including the number of ducts, the number of gland spines and whether they are branched, the length and width of the insect body, and the distance between the median lobes. Overall, our morphological analyses reveal that *P. pentagona* differs significantly from *P. prunicola*. Upon careful examination of the specimens, it is evident that the key distinguishing factor between *P. pentagona* and *P. prunicola* lies in the number and morphology of gland spines. Specifically, *P. pentagona* possesses one gland spine located in the third space on the pygidium. The second, third, or fourth spaces has one bifid or trified gland spine. In contrast, *P. prunicola* exhibits two or more gland spines in the same location. All gland spines are simple and pointed apically, not branched. DNA barcoding was shown to successfully identify scale insects [66]. We used both NJ and ML methods to construct phylogenetic trees based on the COI gene region of *P. pentagona* and *P. prunicola*, and both trees showed the same topology, with different species clustering in separate clades. These results are consistent with previous studies [2,67,68], indicating that the COI gene can be used as a molecular marker for identifying insects from *Pseudaucalaspis*. Since the sample size of scale insects collected for this study was small, we do recommend that further studies incorporate larger sample sizes from additional sites. These new sequences will facilitate the identification of these species in the future and promote phylogenetic studies on the family Diaspididae. Our results also highlight that DNA barcoding appears to be reliable for scale insect species determination [69], although we recommend a more comprehensive molecular phylogenetic study be conducted including more species from the family focusing on additional mitochondrial and nuclear genes to clarify the overall phylogeny of the family.

A niche is the combination of relevant factors and conditions related to the survival of a species. It is composed of environmental factors (such as temperature, humidity, light, resource availability, etc.), the adaptability of the species to extreme environments, the impact of human activity index, intra-specific competition, and other factors. A previous study [12] predicted the potential distribution areas under current and future climate change scenarios based on the known global distribution points of *P. pentagona*, and the results showed that the potential distribution areas were concentrated in southeastern Asia, central Europe, and eastern North America. Bio3 and Bio8 contributed the most to the model modeling, which is consistent with the results of this study. The distribution sites of *P. pentagona* included in the analysis in this study were mainly concentrated in China, and the potential distribution areas were also mainly concentrated in the eastern part of China [12]. In this study, we present the ecological niche model for *P. pentagona* and *P. prunicola*. It is important to note that the practical habitat preference of the two insects may be partially different from our model because climate variables are not the only factors controlling species distribution. Other factors include adaptability to extreme environments, the distribution of hosts, the existence of natural enemies, extreme geographical barriers, and human activities, which will affect the geographical distribution of species [70,71]. However, the influence of climatic conditions on insect growth, development, and reproduction is still considered to be the most important factor in habitat choice and can provide valuable basic information on species distributions [72]. At the same time, we believe in the accurate predictive power provided by the optimized model, which is supported by the analysis of the pROC test.

Compared with other insect families, scale insects have complex feeding habits. It is also predicted that, as a result of climate change, the distribution range of these species will expand, causing more serious damage. They not only endanger landscape plants, but also fruit trees in orchards where they can cause the death of fruit trees in patches resulting in serious economic losses. The potential distribution map constructed by our model can provide a graded reference for pest control. It was suggested that species with similar morphological or phenotypic characteristics may have similar underlying distributions and may be particularly more pronounced in high-suitability areas [73]. For example, in the high-suitability areas to the east of the Hu Huanyong Line in China, farmers need to combine chemical pesticides, biological control, and physical control to remove them. In extreme cases, branches with extremely high densities of insects should be incinerated as the densely stacked shells prevent chemicals from contacting the insects. In regions that have not been infested by insects and are expected to develop into high-suitability areas, government agencies should strengthen quarantine control to prevent large-scale spread through the transportation of seedlings and agricultural products. In moderately suitable areas, the local government should strengthen the detection of insect population densities to prevent large-scale eruptions, thereby preventing devastating economic losses and serious ecological disasters.

In the current study, the environmental constraints on the potential distribution of each species were resolved through a niche model. Environmental variables have a greater impact on the potential distribution of each species, and the biggest limiting factor for *P. pentagona* is Isothermality (Bio3), the second most limiting factor is the mean temperature of the wettest quarter (Bio8). For *P. prunicola*, the biggest limiting factor is the mean temperature of the wettest quarter (Bio8), and the second most limiting factor is the mean diurnal range (Bio2). In the process of species delineation, the most important thing is to distinguish the concept of an ecological species, that is, individuals occupying the same ecological niche or adaptive zone [25,74]. Species delimitation methods are usually divided into tree-based and non-tree-based methods, but none of the currently accepted standards for species delimitation consider spatially specific environmental (climate) information in ecological niches. We argue that niches combined with traditional phylogenetic relationships provide a wealth of information, including visual views of abiotic variables influencing divergence and primary indicators of physiological adaptation. The climatic niches of the two species in this study were quantified and found to be similar but not identical. This supports the morphological and molecular evidence, and the results further underpin the notion that the two species are closely related but distinct.

## 5. Conclusions

We defined two species, *P. pentagona* and *P. prunicola*, based on morphological characteristics, molecular evidence, and ecological niche analysis. The morphological data and molecular phylogenetic analysis of the two species are stable and support a large taxonomic gap between the two. The niche overlap analysis showed that there was some overlap between the two species’ preferences, and the niches were similar but not identical. This indicates that the species are closely related but separate. The study supports the integration of multiple approaches for species delineation including morphological, molecular, and niche evidence.

## Figures and Tables

**Table 1:** Information on the samples used in the molecular study.

Population	Sampling Sites	Host Plant	Species	Date	Lon/Lat
Ingroup					
1	Taigu, Shanxi	*Amygdalus persica*	*P. prunicola*	April 2021	112.55/37.35
2	Yinchuan, Ningxia	*Amygdalus persica*	*P. prunicola*	October 2021	106.26/38.47
3	Linfen, Shanxi	*Amygdalus persica*	*P. prunicola*	September 2021	111.48/36.09
4	Dingbian, Shaanxi	*Cerasus jamasakura*	*P. prunicola*	July 2021	107.5/37.58
5	Taiyuan, Shanxi	*Amygdalus persica*	*P. prunicola*	August 2021	112.5405/37.93527
6	Fuzhou, Fujian	*Aucuba japonica*	*P. pentagona*	July 2021	119.311353/26.066559
Outgroup					
1	Wanning, Hainan	*Trachycarpus fortunei*	*C. aonidum*	June 2022	110.394186/18.803442

**Table 2:** Summary of species sampling locations used for morphometric measurements.

Species	Geographic Origin	Host Plant	Number of Individuals
*P. pentagona*	Fuzhou, Fujian	*Trachycarpus fortunei*	6
	Kunming, Yunnan	*Phoebe zhennan*	5
	Nanning, Guangxi	*Hibiscus mutabilis*	2
	Wuhan, Hubei	*Trachycarpus fortunei*	4
	Dalian, Liaoning	*Fraxinus rhynchophylla*	4
	Shatou, Guangzhou	*Ricinus communis*	3
	Emeishan, Sichuan	*Cycas revoluta*	3
	Emeishan, Sichuan	*Sapium sebiferum*	2
	The Summer Palace, Beijing	*Prunus armeniaca*	4
	Tianjin	*Ailanthus altissima*	1
	Huangyan, Zhejiang	*Ricinus communis*	3
	Hangzhou, Zhejiang	*Morus alba*	3
	Guangzhou, Guangdong	*Morus alba*	2
	Shuyang, Fujian	*Prunus persica*	3
	Taiyuan, Shanxi	*Osmanthus fragrans*	5
*P. prunicola*	Taigu, Shanxi	*Amygdalus persica*	3
	Yinchuan, Ningxia	*Amygdalus persica*	3
	Linfen, Shanxi	*Amygdalus persica*	3
	Dingbian, Shaanxi	*Cerasus jamasakura*	3
	Taiyuan, Shanxi	*Amygdalus persica*	3
	Shanxi Agricultural University	*Prunus davidiana*	3
	Baotou, Neimenggu	*Prunus* spp.	4
	Kunming, Yunnan	*Rhododendron simsii*	3
	Kunming, Yunnan	*Firmiana simplex*	2
	Kunming, Yunnan	*Camellia sinensis*	1
	Kunming, Yunnan	*Sapium sebiferum*	2
	Kunming, Yunnan	*Ligustrum lucidum*	3
	Guangdong Botanical Garden	*Phoebe zhennan*	3
	Huangyan, Zhejiang	*Prunus persica*	5
	Lanzhou, Gansu	*Ulmus pumila*	3
	Chengdu, Sichuan	*Osmanthus*	2
	Hangzhou, Zhejiang	*Prunus* subgen.*Cerasus*	3
	Liaoning	*Syringa oblate* Lindl.	3
	Tianjin	*Cerasus sargentii*	3
	Emeishan, Sichuan	*Prunus davidiana*	2
	Suzhou, Jiangsu	*Prunus mume*	3

**Table 3:** Proportion of variance and principal component loadings of each character on the first two PCs from PCA.

Variables	PC1	PC2
% of variance	25.3%	18.6%
the number of disc pores	0.015436	/
The number of dorsal macroducts in abdomen segment Ⅱ	−0.24286	−0.30082
The number of dorsal macroducts in abdomen segment Ⅲ	−0.25793	−0.29952
The number of dorsal macroducts in abdomen segment Ⅳ	−0.21502	−0.31464
The number of dorsal macroducts in abdomen segment Ⅴ	−0.25672	−0.20047
The number of dorsal macroducts in abdomen segment Ⅵ	−0.03553	−0.09817
The number of perivulvar pores	−0.16445	/
The number of gland spines in the first space	0.044814	−0.04746
First space gland spine branched or not	**0.151556**	−0.25767
The number of gland spines in the second space	0.016068	0.032757
Second space gland spine branched or not	**0.193984**	−0.30188
The number of gland spines in the third space	0.105998	−0.2109
Third space gland spine branched or not	**0.21454**	−0.31626
The number of gland spines in the fourth space	0.087949	−0.32941
Fourth space gland spine branched or not	**0.211909**	−0.28993
Insect body width	−0.22905	−0.09553
Insect body length	−0.2585	−0.09193
The ratio of insect body width: length	0.00973	−0.0246
Distance of anal opening to L1	−0.33608	−0.04948
The ratio of anal opening to L1: insect body length	0.097384	0.088406
L1 width	−0.21788	0.000709
Distance between L1	−0.25517	**0.197385**
The ratio of width between L1: L1 width	−0.16028	**0.266761**
Distance between antennae	−0.21577	0.084799

Remark: Variables in bold indicate greater loading values on each dimension.

**Table 4:** Relative contribution of each environmental variables to MaxEnt model for the two species.

Climatic Variables	*P. pentagona*	*P. prunicola*
Mean diurnal range (Bio2)	11.9	**24.6**
Isothermality (Bio3)	**35.4**	23.1
Mean temperature of wettest quarter (Bio8)	**34.8**	**45.7**
Precipitation seasonality (Bio15)	18.2	4.6

Remark: The first two most-contributing environmental variables are shown in bold.

## Data Availability

The data presented in this study are available on request from all authors.

## Supplementary Materials

The following supporting information can be downloaded at: https://www.mdpi.com/article/10.3390/insects14080666/s1, Figure S1. Performance of niche model under different settings; Figure S2. The results of Partial ROC for two species; Table S1. Distribution sites for *P. pentagona* and *P. prunicola*; Table S2. After filtering distribution sites for *P. pentagona* and *P. prunicola*; Table S3. Pearson correlation coefficients matrix of climatic variables; Table S4. Information used for morphological analyses in this study; Table S5. ENMeval results for *P. pentagona* and *P. prunicola* from SDMs.
