# Supplementary material for: Two Species Delimitation of Pseudaulacaspis (Hemiptera: Diaspididae) Based on Morphology, Molecular Clustering, and Niche Differentiation
Source: Insects. 2023 Jul 25;14(8):666. doi: 10.3390/insects14080666 (PMC10456064; doi:10.3390/insects14080666)

Figure S1. Performance of niche model under different settings.
A: *P. pentagona*; B: *P. prunicola*
Note: L= Linear; Q= Quadratic; H= Hinge; P= Product; T= Threshold.

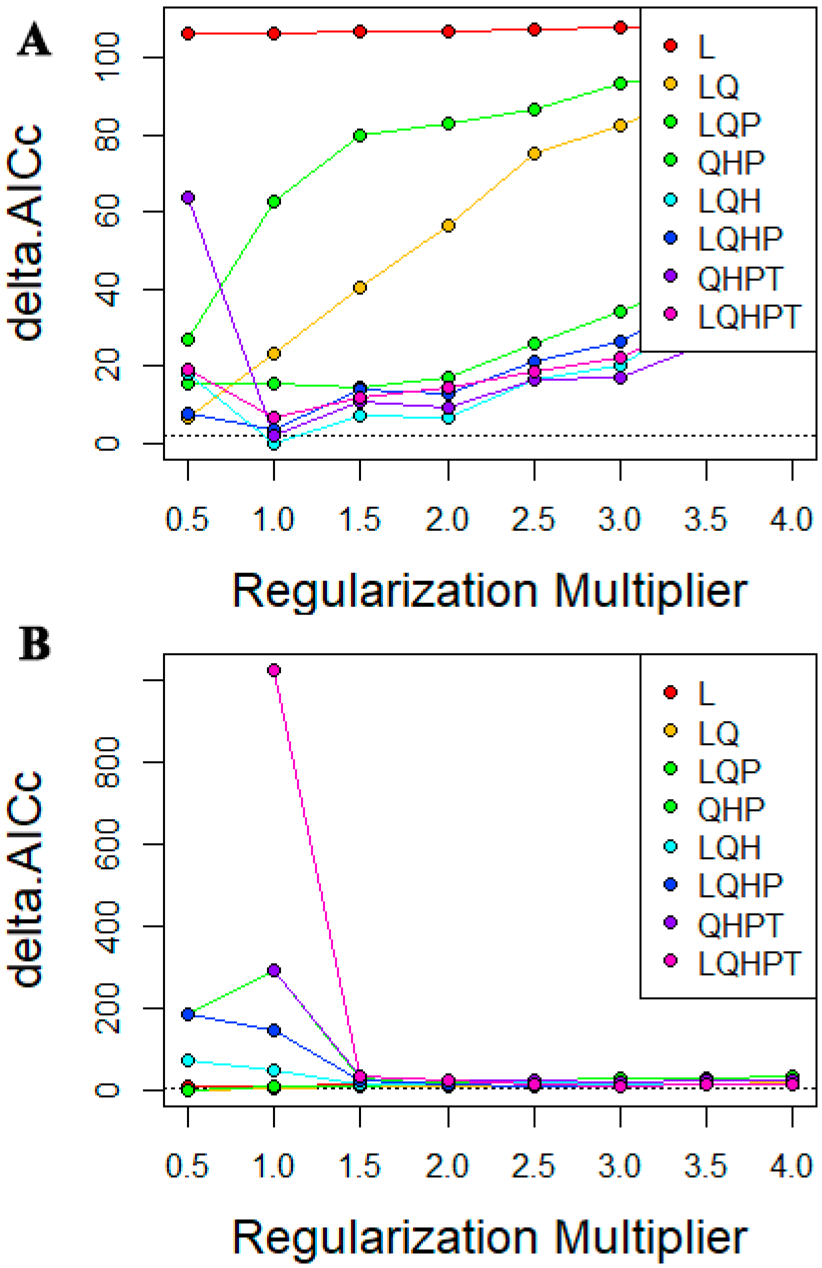

Supplement: Supplementary file 1 [file insects-14-00666-s001.zip › Figure S1.pdf]
