# Supplementary material for: Two Species Delimitation of Pseudaulacaspis (Hemiptera: Diaspididae) Based on Morphology, Molecular Clustering, and Niche Differentiation
Source: Insects. 2023 Jul 25;14(8):666. doi: 10.3390/insects14080666 (PMC10456064; doi:10.3390/insects14080666)

Figure S2. The results of Partial ROC for two species.

Note: Partial AUC distribution for two species generated through 1000 bootstrap iterations with 5% omission in the ROC space. Higher partial AUC ratios indicate the better predictive ability of the Maxent model.

A: *Pseudaulacaspis pentagona*

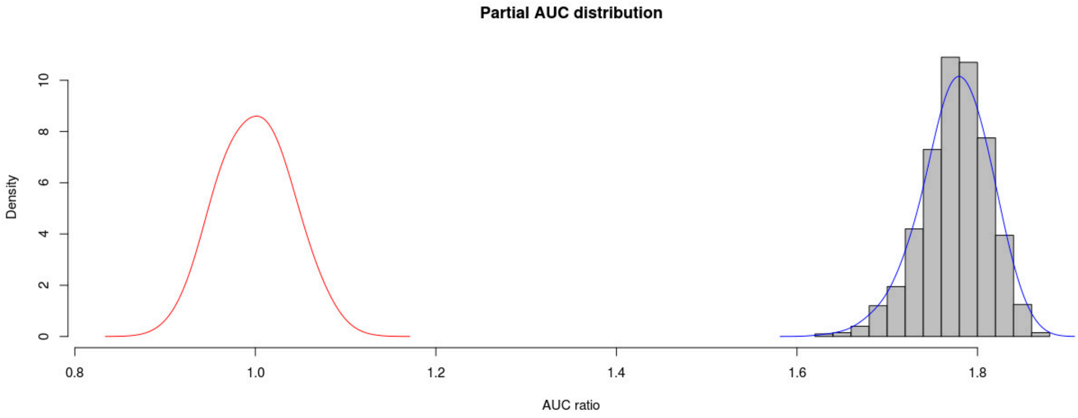

B: *Pseudaulacaspis prunicola*

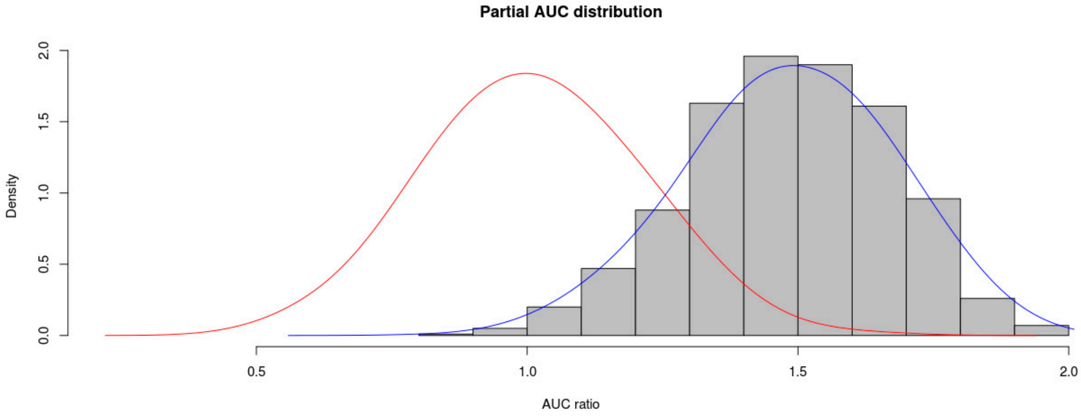

Supplement: Supplementary file 1 [file insects-14-00666-s001.zip › Figure S2.pdf]
