# Supplementary material for: Two Species Delimitation of Pseudaulacaspis (Hemiptera: Diaspididae) Based on Morphology, Molecular Clustering, and Niche Differentiation
Source: Insects. 2023 Jul 25;14(8):666. doi: 10.3390/insects14080666 (PMC10456064; doi:10.3390/insects14080666)
Supplement: Supplementary file 1 [file insects-14-00666-s001.zip › Table S3.pdf]

Table S3 Pearosn correlation coefficients matrix of climatic variables

**1**	1																		
**2**	-.772**	1																	
**3**	.226	.226	1																
**4**	-.801**	.576**	-.649**	1															
**5**	.490**	-.344**	-.488**	.105	1														
**6**	**.974****	-.828**	.279*	-.884**	.316**	1													
**7**	-.870**	.762**	-.451**	**.966****	-.012	**-.952****	1												
**8**	.722**	-.645**	-.210	-.301*	.669**	.632**	-.451**	1											
**9**	**.965****	-.780**	.280*	-.855**	.366**	**.976****	**-.911****	.614**	1										
**10**	.756**	-.658**	-.367**	-.216	**.914****	.631**	-.371**	.841**	.649**	1									
**11**	**.970****	-.741**	.400**	**-.922****	.272*	**.989****	**-.955****	.587**	**.973****	.576**	1								
**12**	.865**	-.826**	.053	-.713**	.384**	.886**	-.810**	.561**	.874**	.646**	.853**	1							
**13**	.734**	-.738**	.091	-.652**	.209	.771**	-.746**	.493**	.712**	.491**	.739**	.890**	1						
**14**	.711**	-.726**	-.207	-.423**	.576**	.691**	-.543**	.498**	.747**	.719**	.641**	.852**	.591**	1					
**15**	-.633**	.598**	.070	.464**	-.454**	-.636**	.524**	-.330**	-.705**	-.555**	-.608**	-.688**	-.305*	-.854**	1				
**16**	.788**	-.741**	.179	-.734**	.193	.828**	-.810**	.490**	.774**	.487**	.807**	**.922****	**.984****	.623**	-.389**	1			
**17**	.711**	-.725**	-.215	-.416**	.587**	.688**	-.536**	.495**	.739**	.726**	.637**	.859**	.603**	**.996****	-.851**	.632**	1		
**18**	.726**	-.690**	.233	-.730**	.076	.779**	-.796**	.508**	.719**	.387**	.765**	.851**	.955**	.505**	-.277*	**.966****	.510**	1	
**19**	.689**	-.697**	-.232	-.386**	.605**	.661**	-.502**	.480**	.734**	.726**	.612**	.819**	.555**	.974**	-.839**	.580**	**.972****	.449**	1
	**1**	**2**	**3**	**4**	**5**	**6**	**7**	**8**	**9**	**10**	**11**	**12**	**13**	**14**	**15**	**16**	**17**	**18**	**19**
